# A Rare Coexistence of Villoglandular Papillary Adenocarcinoma of the Uterine Cervix and Brenner Tumor of the Ovary

**DOI:** 10.1155/2014/342040

**Published:** 2014-02-23

**Authors:** Kadir Guzin, Sadik Sahin, Gokhan Goynumer, Mustafa Eroğlu, Akın Usta, Nurver Ozel

**Affiliations:** ^1^Medeniyet University of Istanbul, Goztepe Education and Research Hospital, Clinics of Gynecology and Obstetrics, Istanbul, Turkey; ^2^Zeynep Kamil Women's and Children's Hospital, Department of Gynecology, Op. Dr. Burhanettin Üstünel Caddesi, No. 10, 34668, Üsküdar, 34722 Istanbul, Turkey; ^3^Fatih Sultan Mehmet Training and Research Hospital, Department of Pathology, Istanbul, Turkey

## Abstract

Synchronous primary gynecological cancers have been reported to be seen rarely in the literature. In this report, we aimed to describe a 51-year-old patient with the coexistence of villoglandular papillary adenocarcinoma of the cervix uteri and Brenner tumor in the right ovary. She successfully underwent radical hysterectomy, bilateral salphing-oopherectomy and pelvic and para-aortic lymphadenectomy.

## 1. Introduction

The coexistence of carcinomas of the cervix and ovary has been reported to be a rare situation in gynecological practice. It is often unclear whether this represents synchronous primary tumors or metastasis that consequently alters staging, management, and expected outcome of these patients. In a study by Kaminski and Norris [1], the incidence of concomitant ovarian cancers (both primary and metastasis) in patients with cervical carcinoma was reported to be as high as 16% and only 9% of those cases were primary ovarian tumors. In the same study, presence of the endometrioid type adenocarcinoma of the cervix was found to dictate the presence of the primary independent ovarian tumour. Villoglandular papillary adenocarcinoma (VPA) is a very rare subtype of adenocarcinoma of the uterine cervix. It has a good prognosis and generally occurs in women of child bearing age [2]. Although the etiology has not been well understood, oral contraceptive use and infection with human papilloma virus (HPV) are being suggested as the etiological factors. Our purpose in this case is to report a patient with a rare coexistence of cervical VPA and Brenner tumour of the ovary.

## 2. Case Description

**Figure 1:**
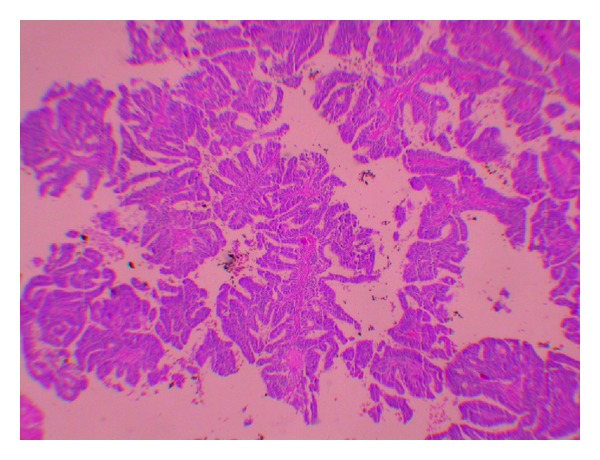
Appearance of the papillae in villoglandular papillary adenocarcinoma of the uterine cervix (H&E stain, ×40).

**Figure 2:**
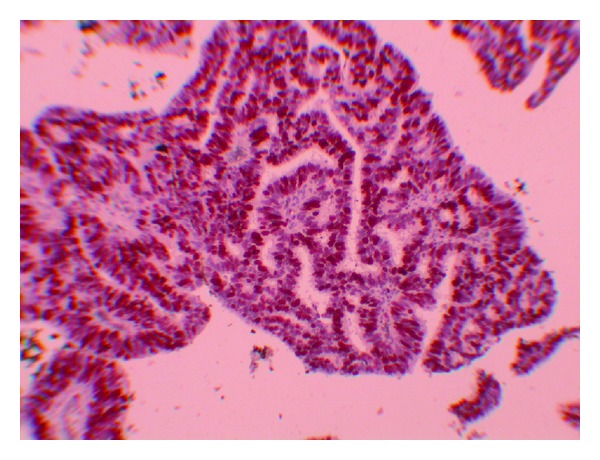
Appearance of the papillae in villoglandular papillary adenocarcinoma of the uterine cervix immunohistochemical staining (IH).

**Figure 3:**
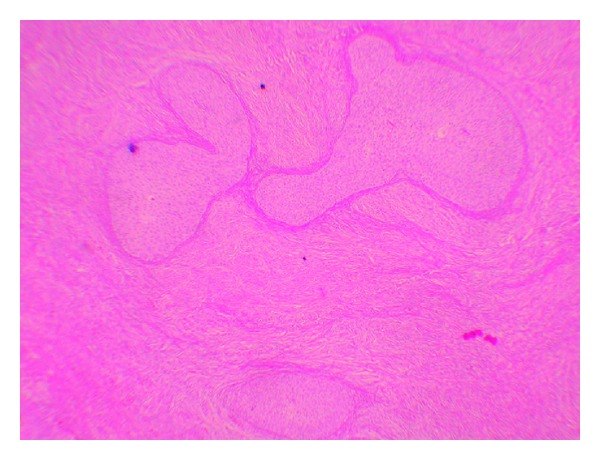
Appearance of Brenner tumor of ovary (hematoxylin and eosin staining (H&E)).

A 51-year-old female (gravida 3, parity 2) was admitted to the medical center after experiencing vaginal discharge and spotting for about 2 months. Approximately, 2 years prior to being admitted she had visited a different medical center and a pap smear test had been performed and exfoliated cells resulted in the diagnosis of atypical glandular cells of undetermined significance (AGUS). Following this diagnosis, it was recommended to the patient to undergo colposcopy and biopsy. But the patient failed to return. Her medical history was otherwise unremarkable. Her gynecological examination revealed an approximately 3 × 2 cm vegetative polypoid lesion originating from the endocervical canal and protruding into the vagina. A transvaginal ultrasound revealed an endometrial thickness of 4 mm and a right ovarian solid mass measuring 25 × 21 mm. Laboratory study revealed that cancer antigen (CA) 125 was 9.46 U/mL, CA15.3 was 8.6 U/mL, CA19.9 was 4.75 U/mL, and carcinoembryonic antigen (CEA) was 1.4 U/mL. A cervical punch biopsy was performed and “villoglandular papillary cervical adenocarcinoma” was documented. Following the biopsy procedure, a radical abdominal hysterectomy, a bilateral salpingo-oophorectomy, and a pelvic and para-aortic lymphadenectomy were performed. The hysterectomy specimen revealed an exophytic, polypoid mass with a diameter of 2.5 × 2 × 2 cm occupying the posterior of the cervix and protruding into the vagina. Histologically, the cervical tumor was composed of large cystic glandular and papillary structures surrounded by stroma resembling those of the normal cervix (Figure 1). Mild to moderate cytological atypia was present with some variation in nuclear size and shape. Three to four mitotic figures per 10 high-power fields were present in the epithelial cells. No invasion focus of the atypical small gland in the wall of the cervix was noticed. There was no lymph node invasion. The final pathology was reported as “cervical papillary adenocarcinoma” and The International Federation of Gynecology and Obstetrics (FIGO) staging was defined as stage IBI. Immunohistochemical staining for HPV types 16 and 18 in the cervical epithelial cells was found negative (Figure 2). The right ovarian examination revealed a Brenner tumor with sharply demarcated epithelial nests in a dense fibrous stroma (Figure 3). The epithelial cells were relatively uniform in size with prominent cell borders and a pale to eosinophilic cytoplasm. Atypia and mitotic activity were not seen. The stroma was densely fibrous and contained hyalinized areas and dystrophic calcification. Immunoperoxidase staining for HPV types 16 and 18 in the cervical neoplastic epithelial cells was found negative. She received no adjuvant therapy and the 12-month follow-up showed no evidence of recurrent disease.

## 3. Discussion

Synchronous tumors are defined as two or more tumors occurring in a patient simultaneously. The occurrence rate of synchronous gynecologic malignancies varies between 0.7 and 1.8% in patients with gynecologic tumors. The common occurrence of synchronous ovarian and endometrial adenocarcinoma is especially well known, due to the hormonally active nature of the tumors of the ovary [3].

Villoglandular papillary adenocarcinoma (VPA) is a very rare subtype of adenocarcinoma of the uterine cervix, but a well recognized variant of cervical adenocarcinoma with a favorable prognosis and generally occurring in women of child bearing age [4]. Many of the cases of VPA and pregnancy have been reported in the literature. However, none of them had a combination with Brenner tumor of the ovary. Brenner tumors are known to be a rare ovarian neoplasm and they are generally monolateral, more rarely bilateral, and often associated with endometrial disorders related to oestrogenic production [5].

The collected data in the present case support that multiple neoplasms with different embryological and histological origin might occur in female genital tract simultaneously. It is important to distinguish multiple primary neoplasms from metastatic disease because of the fact that overall survival as well as treatment would vary considerably. Synchronous primary genital tumors generally have better prognoses than tumors of single metastatic lesions as they tend to be diagnosed at early stages and at low grades [6]. It should be noted, however, that concurrent cervical-ovarian tumors are usually detected in younger and leaner premenopausal women who smoke.

In our patient, a radical abdominal hysterectomy, a bilateral salpingo-oophorectomy, and a pelvic and para-aortic lymphadenectomy were performed. If Brenner tumors were diagnosed to be malignant tumors, an omentectomy would be added during surgery and chemotherapy and radiation therapy would also be necessary postoperatively.

In the literature, there is still a debate about whether concomitant tumors are independent primary tumors or micrometastasis. In a study by Elishaev et al., it was clearly reported that endocervical adenocarcinomas, including some qualifying as microinvasive, can metastasize to the ovaries and simulate primary ovarian surface epithelial neoplasms [6]. Therefore the authors suggest that detection of HPV DNA in these ovarian tumors should be performed to differentiate metastatic endocervical adenocarcinomas from independent primary tumours of the ovary. The important role of immunohistochemical analysis for p16 and/or HPV analysis by in situ hybridization or polymerase chain reaction (PCR) is recommended to identify such cases [7].

In our patient, immunoperoxidase staining for HPV types 16 and 18 in the cervical epithelial cells was found negative. Because of the benign nature of the Brenner tumour, we did not perform either immunohistochemistry staining for HPV or PCR in the ovarian tissue.

In conclusion, concurrent occurrence of cervical villoglandular adenocarcinoma and Brenner tumor of the ovary is an exceptionally rare situation. To differentiate the nature of the concurrent ovarian tumour, either primary or micrometastasis, from cervical adenocarcinoma, HPV immunohistochemistry or DNA sampling should be performed in a routine manner especially for malignant concurrent ovarian tumours in addition to the pathological examination.
